# Exogenous Melatonin Induces Salt Stress Tolerance in Cucumber by Promoting Plant Growth and Defense System

**DOI:** 10.3390/life15081294

**Published:** 2025-08-14

**Authors:** Guangchao Yu, Zhipeng Wang, Ming Wei, Lian Jia, Yue Qu, Yingyi Jiang, Shihan Xiang

**Affiliations:** 1 College of Chemistry and Life Sciences, Anshan Normal University, Anshan 114007, China; wangzp1326@gmail.com (Z.W.); wm050820@163.com (M.W.); jl_58@163.com (L.J.); quyue199209@163.com (Y.Q.); jiangdi031507@163.com (Y.J.); xsh20050701@163.com (S.X.); 2 Liaoning Key Laboratory of Development and Utilization for Natural Products Active Molecules, Anshan Normal University, Anshan 114007, China

**Keywords:** cucumber, melatonin, salt stress, antioxidant enzymes, biomass, salt stress response genes

## Abstract

This study aims to investigate the regulatory effect of exogenous melatonin (MT) on the growth and development of cucumbers subjected to salt stress. Using the XinTaiMiCi material and indoor pot culture method, seven treatments were set up: control group (CK), T0 (salt treatment group, 150 mM S + 0 μM MT), T1 (150 mM S + 25 μM MT), T2 (150 mM S + 50 μM MT), T3 (150 mM S + 100 μM MT), T4 (150 mM S + 150 μM MT), and T5 (150 mM S + 200 μM MT). Changes in plant height, stem diameter, leaf area, relative chlorophyll content, antioxidant enzyme activity, reactive oxygen species content, and osmotic adjustment substance content in cucumber seeds and seedlings under different treatments were studied, and a correlation analysis of these indicators was conducted. Meanwhile, the expression of salt stress-related genes was detected in all seven treatment groups. The results showed that, compared to the CK, T0 significantly reduced the hypocotyl length, root length, hypocotyl diameter, root diameter, and fresh and dry weights of cucumber seeds; in the later stage of salt stress treatment, T0 significantly increased the activities of superoxide dismutase (SOD), peroxidase (POD), catalase (CAT), and malondialdehyde (MDA) and the content of soluble protein in seeds. Additionally, T0 significantly increased the plant height, root length, stem diameter, leaf area, and fresh and dry weights of cucumber seedlings per plant; in the later stage of salt stress treatment, T0 significantly increased the activities of SOD, POD, CAT, and MDA and the content of soluble protein and chlorophyll in leaves. Compared to T0, the application of 50 μmol·L^−1^ MT under salt stress significantly increased the plant height, stem diameter, root length, leaf area, and fresh and dry weights of cucumber seedlings per plant; significantly increased the activities of SOD, POD, and CAT; decreased the MDA activity; and significantly increased the content of soluble protein and chlorophyll. Under salt stress conditions, the exogenous application of low-concentration melatonin increased the expression levels of salt stress response genes (such as *CsSOS*, *CsNHX*, *CsHSF,* and *CsDREB*) in cucumber. The germination rate (GR), germination potential (GP), germination index (GI), plant height (PH), root length (RL), leaf area index (LAI), fresh weight (FW), dry weight (DW), soluble protein (SP), relative chlorophyll content (SPAD), POD, CAT, and SOD of cucumber seedlings exhibited significant positive correlations, whereas they were negatively correlated with MDA content. In conclusion, the application of 50 μM MT can effectively alleviate the oxidative and osmotic stress caused by a high-salt environment in cucumber, promote cucumber growth, and improve salt tolerance.

## Simple Summary

This study investigates the mechanism by which exogenous melatonin (MT) mitigates the effects of salt stress on the growth and development of cucumber. The optimal concentration of exogenous MT treatment significantly alleviated the damage caused by salt stress, promoting seed germination and growth; increasing plant height, root length, stem diameter, leaf area, and the fresh and dry weights of cucumber seedlings; enhancing chlorophyll content; and inhibiting the excessive production of oxidative stress markers. Simultaneously, exogenous MT significantly enhanced the expression of salt stress-related genes (*CsSOS*, *CsNHX*, *CsHSF*, and *CsDREB*), thereby improving the plant’s stress resistance level. In summary, 50 μM MT can effectively alleviate the oxidative and osmotic stress caused by high-salt environments on cucumber, promote cucumber growth, and enhance salt tolerance.

## 1. Introduction

Soil salinization has become a significant ecological issue threatening the sustainable development of global agriculture. Due to the combined effects of population expansion, excessive industrial wastewater discharge, and extensive irrigation management, the area of salinized farmland is rapidly expanding, severely affecting plant growth and development [1]. Salt stress exerts multi-stage physiological inhibitory effects on plants, such as hindering seed water absorption and expansion, inhibiting growth, disrupting floral organ development, and resulting in decreased crop yield and quality [2,3]. Studies have found that excessive sodium ions (Na^+^) and chloride ions (Cl^−^) in saline environments can trigger ion toxicity effects in plant cells, ultimately leading to significantly reduced seed germination rates [4]. Salt stress inhibits seedling growth, and high-salt concentrations in the soil decrease the efficiency of water absorption by roots, causing osmotic stress and ultimately leading to a decrease in the relative water content of leaves, as well as slow plant growth [5]. The water deficit induced by salt stress can lead to a decrease in stomatal conductance in plants, thereby inhibiting photosynthetic efficiency and promoting excessive accumulation of reactive oxygen species (ROS). These highly reactive oxygen molecules, including hydrogen peroxide (H_2_O_2_), singlet oxygen (^1^O_2_), superoxide anion (O_2_•^−^), and hydroxyl radical (•OH), are highly oxidizing and ultimately destroy the integrity of plant cell structures [6]. Therefore, plants alleviate oxidative damage mediated by ROS by activating their endogenous antioxidant defense systems. Superoxide dismutase (SOD), peroxidase (POD), and catalase (CAT) are enzymatic cascade reaction substances that can effectively scavenge free radicals and maintain redox homeostasis, thereby enhancing plant tolerance to adversity [7]. Malondialdehyde (MDA) in plants is the core end product of membrane lipid peroxidation, and its content directly reflects the degree of cell membrane damage. Salt stress induces a burst of reactive oxygen species (ROS), triggering a chain reaction of membrane lipid peroxidation in plants. MDA accumulates as the final toxic product, leading to increased membrane permeability, decreased fluidity, and disruption of membrane structural integrity [8]. Studies have found that antioxidant enzyme activity in cowpea seedlings increased in response to elevated salt concentrations under varying NaCl treatments [9]. When rice is exposed to NaCl stress, its antioxidant enzyme activity exhibits an initial increase followed by a decrease in the middle and later stages, reflecting a balance between short-term defense and long-term damage [10]. In response to 150 mmol·L^−1^ NaCl, the physiological and growth-related traits of eggplant seedling leaves exhibited the most pronounced decline, while the enzymatic activities of ascorbate peroxidase (APX), CAT, SOD, POD, and glutathione reductase (GR) demonstrated significant enhancements [11]. Studies have found that moderate stress with 0.3% (mass fraction) NaCl can stimulate an increase in the content of soluble sugars and soluble proteins in rice roots [12]. In summary, to resist salt stress damage, plants build a physiological protective barrier through the accumulation of osmoregulatory substances (soluble proteins), antioxidant enzyme systems (such as SOD and POD), markers of membrane lipid peroxidation (MDA), and photosynthetic pigments (chlorophyll).

Melatonin (MT), a hormone belonging to the indoleamine class, is synthesized from tryptophan. It plays a significant role in crops’ resistance to adverse environmental stresses [13]. As an endogenous substance in plants, melatonin is categorized as a novel plant growth regulator, playing a crucial role in enhancing crops’ resistance to abiotic stresses such as drought and saline-alkali conditions. Studies have found that melatonin can effectively alleviate the damage to plant cells caused by oxidative stress by enhancing the reactive oxygen species (ROS) scavenging system within plants. Additionally, it can regulate the synthesis of osmotic adjustment substances, thereby maintaining the water balance of plant cells under adverse conditions [14]. Chen et al. showed that applying 100 μmol·L^−1^ melatonin to corn significantly increased chlorophyll content in its leaves under salt stress, enhanced antioxidant enzyme activity, and ultimately alleviated oxidative damage caused by salt stress, thereby enhancing plant salt tolerance [15]. Under 150 mmol·L^−1^ NaCl conditions, 10 μmmol·L^−1^ MT treatment increased the content of soluble sugars and soluble proteins in cotton roots, maintaining cell integrity and alleviating the inhibitory effect of salt stress on cotton root development [16]. We found that melatonin seed priming with 500 µM MT stabilized the chloroplast structure in maize seedlings under drought stress [17]. Li et al. discovered that melatonin treatment significantly improved the photosynthetic efficiency of alfalfa seedlings under salt stress [18]. Under saline-alkali stress conditions, the photosynthetic system of crops can be significantly inhibited [18]. Exogenous melatonin effectively alleviates this phenomenon through multiple protective mechanisms. It can maintain the integrity of the chloroplast ultrastructure, maintain a certain level of chlorophyll content, and activate and enhance the accumulation of antioxidant systems and osmotic adjustment substances, thereby enhancing the photosynthetic capacity of crops.

Currently, research on the effects of slow-release melatonin on cucumber salt stress is still limited. However, existing studies have shown that salt stress significantly affects the physiological responses of rice and Arabidopsis thaliana. Additionally, salinity can interfere with crop osmotic regulation and ion signaling pathways [19]. Among them, the salt overly sensitive (SOS) pathway has been confirmed as a key mechanism for maintaining cellular ion homeostasis and responding to salt stress [20]. OsNHX1 (Na^+^/H^+^) exchanger, as an important transcription factor, alleviates salt damage by regulating the concentration of Na^+^ and K^+^ in rice plants under NaCl stress [21]. Studies have also found that exogenous melatonin can significantly upregulate the expression levels of stress response genes (such as *OsSOS*, *OsNHX*, *OsHSF*, and *OsDREB*) in rice under salt stress [22]. Furthermore, Most of the AetHsfs were found to be upregulated by heat stress, while some also showed expression in response to drought, salinity, and high light stress [23].

Cucumber (*Cucumis sativus* L.) is one of the important economic crops in China. The roots of cucumber are relatively delicate, and high concentrations of salt can lead to a decrease in cucumber yield and quality. Therefore, the phenomenon of secondary salinization of soil has become one of the biggest obstacles affecting the yield of protected cucumber cultivation [24]. To mitigate the threat of salinization to the agricultural industry, improve the utilization rate of saline soil, and maintain cucumber quality, researchers have conducted studies on the salt tolerance of cucumber. Currently, research on the effects of melatonin (MT) on cucumber under salt stress mainly focuses on seed germination and seedling growth [25], with fewer reports on the regulatory effects of MT on later stages of cucumber growth and development under salt stress. Therefore, this study focuses on the specific effects of exogenous melatonin on cucumber seed germination, plant morphology, biomass, and physiological and biochemical indicators of leaves during the growth period of cucumber under salt stress. The aim is to provide scientific evidence and practical guidance for enhancing cucumber cultivation performance in saline environments through the use of exogenous melatonin.

## 2. Materials and Methods

### 2.1. Plant Materials

The cucumber material used for the experiment was XinTaiMiCi. It was grown in the soil culture room at Anshan Normal University. MT was purchased from Sigma Company and prepared into a 200 mmol·L^−1^ stock solution with 95% (*v*/*v*) ethanol, which was stored in a −20 °C refrigerator. When in use, it was diluted to the corresponding concentration of MT solution.

### 2.2. MT Concentration Screening

Select plump cucumber seeds for experimentation and disinfect them before the experimental treatment. Take a glass Petri dish with a diameter of 120 mm, lay two layers of filter paper inside as a germination bed, and place it in a constant temperature incubator (28 °C) for germination for 1 day. Select seeds with consistent sprouting from the seedling tray and place them in the Petri dish. Lay two layers of filter paper at the bottom of each Petri dish and place 60 seeds in each.

Seeds of sowing cucumber with a consistent degree of seed coat exposure were selected and planted in pots. The cultivation was carried out in the soil culture room of the Key Laboratory of Natural Products Research, Anshan Normal University. The indoor temperature ranged from 28 °C during the day to 25 °C at night, with a 14 h light and 10 h dark cycle, and the relative humidity was maintained at 45% to 50%.

After the cucumber seedlings have developed one true leaf, start the treatment for each seedling.

In this experiment, a total of seven groups of cucumber plants were involved. Each group consisted of three replicates, with five plants in each replicate. The experimental groups were designated as the control group (CK), T0 (salt treatment group, 150 mM S + 0 μM MT), T1 (150 mM S + 25 μM MT), T2 (150 mM S + 50 μM MT), T3 (150 mM S + 100 μM MT), T4 (150 mM S + 150 μM MT), and T5 (150 mM S + 200 μM MT). One week prior to salt stress exposure, plants were treated with melatonin every other day, as described in Reference 40 and our preliminary screening. For salt stress, plants were treated with 150 mM NaCl every 2 days, with four consecutive treatments. In the experiment, fully expanded young leaves were randomly selected from each experimental group. Additionally, a total of seven groups of cucumber seeds were involved. Each group consisted of three replicates, with 10 cucumber seeds in each replicate, and the treatment gradient was the same as above. Growth and physiological indicators were measured for cucumber seeds on the 5th day of salt treatment and the 25th day after treatment.

### 2.3. Morphological Index Determination

The distance from the cotyledon node to the apical growth point of the cucumber was measured vertically to obtain the plant height. The stem diameter was measured 1 cm above the cotyledon node using a vernier caliper. Leaf area = leaf length × leaf width × 0.75 [26].

### 2.4. Determination of Fresh and Dry Matter Masses

Fresh and dry matter masses of cucumber: After 28 days of salt stress treatment, the fresh matter mass of the entire cucumber seedling was obtained. Then, it was placed in an oven, first blanched at 105 °C for 30 min, and then dried at 80 °C for 72 h until the mass remained constant. The dry matter mass was then measured. Cucumber seeds were collected 5 days after treatment for measurements of both fresh and dry weights.

### 2.5. Determination of Physiological and Biochemical Indicators

For each treatment, three cucumber seedlings were selected, wrapped in tin foil, flash-frozen using liquid nitrogen, and stored in a −80 °C refrigerator. The total superoxide dismutase (SOD) [27], peroxidase (POD) [28], and catalase (CAT) [29] activities of the samples were measured using the total superoxide dismutase assay kit, peroxidase assay kit, and catalase assay kit from Nanjing Jiancheng Biological Engineering Research Institute, respectively. The soluble protein content was determined using a total plant protein assay kit, and the malondialdehyde (MDA) content was measured using an MDA assay kit. Relevant physiological indicators of cucumber seeds were detected using the aforementioned kits [30].

### 2.6. Chlorophyll Contents

Select 0.1 g of leaves, cut them into pieces, and place them in a 10 mL centrifuge tube containing 5 mL of 95% ethanol. Treat them in the dark for 24 h and then measure their absorbance at wavelengths of 645 nm and 663 nm [31].

### 2.7. Quantitative Real-Time RT-qPCR

Total RNA was extracted from the cucumber samples using the RNAprep Pure Plant Kit (TianGen Biotech, Beijing, China) according to the manufacturer’s instructions. The isolated RNA was subsequently reverse transcribed into complementary DNA (cDNA) using the FastQuant RT Kit (with gDNase) (KR106). Real-time quantitative PCR (qPCR) was conducted employing the SuperReal PreMix Plus Kit (SYBR Green) (FP205) for fluorescence detection. The specific primers targeting candidate genes were designed and analyzed via the online tool available at https://quantprime.mpimp-golm.mpg.de/ (accessed on 28 April 2025). The RT-qPCR reaction volume was 10 μL, consisting of 1 μL of cDNA template, 4.5 μL of 2 × SuperReal PreMix Plus, 0.1 μL of forward (F) primer, 0.1 μL of reverse (R) primer, and 4.3 μL of nuclease-free water. The reaction was incubated at 95 °C for 15 min, followed by forty cycles at 95 °C for 10 s, 60 °C for 20 s, and 72 °C for 1 min, respectively. The melting curve analysis stage was conducted at 95 °C for 5 s, 60 °C for 1 min, and 72 °C for 30 s. We selected the internal reference gene as the cucumber actin gene [32]. Supplementary Table S1 shows the specific primers.

### 2.8. Data Processing and Analysis

Data are the mean ± standard deviation from three biological replicates per cultivar. Standard deviation was assessed using Excel. Statistical significance was analyzed using Student’s *t*-test (*p* < 0.05 or *p* < 0.01) through SPSS 27.0.1 software. Origin 2021 was used for correlation analysis.

## 3. Results

### 3.1. The Effect of Exogenous MT on Cucumber Seed Germination Under Salt Stress

**Figure 1:**
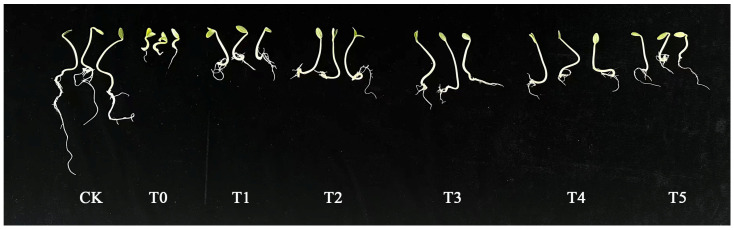
Effects of different concentrations of exogenous MT (Melatonin) on germination and growth of cucumber seeds under salt stress. CK: Control group (distilled water); T0: 150 mM salt treatment; T1: S + 25 μM MT group; T2: S + 50 μM MT group; T3: S + 100 μM MT group; T4: S + 150 μM MT group; T5: S + 200 μM MT group; S: 150 mM salt treatment.

Phenotypic observations of cucumber seeds grown for 5 days under salt stress with different concentrations of melatonin added are shown in Figure 1. Compared with the control and cucumber seeds under salt stress alone, melatonin solutions at concentrations of 25 μM, 50 μM, 100 μM, and 150 μM had varying degrees of promoting effects on the growth of cucumber seed roots. Compared with the CK, the hypocotyl length and root length in the sole salt treatment group (T0) were significantly reduced, indicating that salt stress significantly decreased the hypocotyl length and root length of cucumber seeds during the germination period (Table 1). Compared with the T0 group, the hypocotyl length and root length of the other treatment groups were significantly increased, indicating that exogenous addition of MT could significantly increase the hypocotyl length and root length. T3 was significantly higher than the other treatments, suggesting that spraying 100 μM MT could significantly alleviate the inhibitory effect of salt stress on the hypocotyl length of cucumber seeds during the germination period. As shown in Table 1, compared with the CK, the hypocotyl thickness of each treatment group was inhibited. However, compared with T0, the inhibition of hypocotyl thickness in the T1, T2, T3, and T4 treatment groups was alleviated. Among them, T3 showed the most significant alleviation in hypocotyl thickness, which was consistent with that of the CK. The hypocotyl thickness of the T5 treatment group did not change significantly compared with that of the T0 group, indicating that exogenous MT can significantly increase hypocotyl thickness under salt stress. The alleviating effect of T3 on salt stress was significantly higher than that of other treatments. Statistical analysis of root thickness data revealed that T3 showed the most obvious alleviation of root thickness, with no significant difference from the CK, but significantly higher than the other treatments. Compared with T0, there was no significant difference in root thickness between the T1, T2, and T5 treatment groups, while the root thickness of the T3 and T4 treatment groups was significantly increased. Thus, it can be concluded that salt stress significantly inhibits the growth of hypocotyl and root thickness in cucumber seeds during the germination period. The exogenous addition of an appropriate concentration of MT can alleviate the inhibitory effect of salt on root thickness in cucumber seeds during the germination period, thereby promoting root growth. The MT concentration in T3 had a more significant alleviating effect on salt stress than the other treatments. As shown in Table 1, compared to the CK, the fresh weight of the T0 treatment group was significantly reduced, indicating that salt stress reduced the fresh and dry weights of cucumber seeds during the germination period. Compared with the T0 treatment group, the fresh weight and dry weight of the other treatment groups increased, with the T3 treatment group showing the most significant increase trend. These findings demonstrate that through the analysis of phenotypic characteristics and growth indicators during cucumber seed germination, a melatonin concentration of 100 μM MT (T3) exhibits the most pronounced effect in alleviating the inhibitory impact of salt stress on seed germination, with 50 μM MT (T2) melatonin showing a comparatively lesser but still notable effect.

### 3.2. The Effects of Different Concentrations of MT on the Growth Indices of Cucumber Seedlings Under Salt Stress

**Figure 2:**
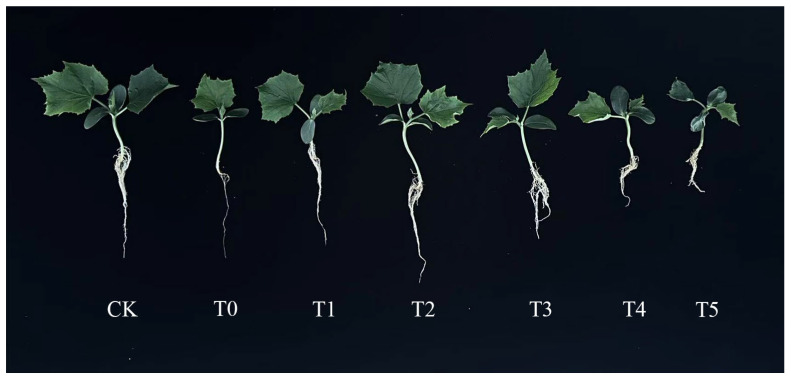
Effects of different concentrations of exogenous MT (Melatonin) on the growth and development of cucumber seedlings under salt stress. CK: Control group (distilled water); T0: 150 mM salt treatment; T1: S + 25 μM MT group; T2: S + 50 μM MT group; T3: S + 100 μM MT group; T4: S + 150 μM MT group; T5: S + 200 μM MT group; S: 150 mM salt treatment.

When the cucumber seedlings had fully developed true leaves and immature young leaves, salt stress and a melatonin slow-release solution were applied. After 25 days of cultivation, the growth status of the cucumber seedlings was observed. The phenotype of the cucumber seedlings is shown in Figure 2. Compared with the control group (CK), the number of leaves and lateral roots in the salt stress (T0) group decreased, indicating that the growth of cucumber seedlings under salt stress was significantly inhibited. After adding different concentrations of melatonin, the root length, number of lateral roots, growth status, and number of leaves in the T2 treatment group were generally consistent with those in the control, indicating that the melatonin concentration in the T2 treatment group completely alleviated the salt stress on cucumber seedlings. The concentrations of melatonin in the T1 and T3 treatment groups could partially relieve the salt stress on cucumber seedlings. At the same time, the concentrations of melatonin in the T4 and T5 treatment groups were too high, inhibiting the growth of cucumber seedlings under salt stress.

As shown in Table 2, compared to the control group (CK), the T0 group significantly reduced the plant height, root length, and stem diameter of cucumber seedlings (*p* < 0.05). The plant height, root length, and stem diameter decreased by 30.7%, 11.6%, and 43.3%, respectively, indicating that salt stress significantly inhibited the growth of cucumber seedlings. Under 150 mM NaCl stress treatment, with the increase in MT concentration, the plant height, root length, and stem diameter of cucumber seedlings showed a trend of first increasing and then decreasing. In terms of plant height, the T2 and T3 treatment groups recovered to a level not significantly different from the CK, indicating that melatonin at an appropriate concentration could completely reverse the negative effects of salt stress. Compared to T0, the low-concentration T1 group and the high-concentration T4 and T5 groups had limited mitigating effects on the growth of cucumber seedlings under salt stress. In terms of root length, the T0 group had a significantly lower root length than the control group, with a decrease of 11.6%, indicating that salt stress disrupted the physiological balance of root development. Compared to T0, the root lengths of T1 and T2 significantly increased (*p* < 0.05) by 12.39% and 21.58%, respectively. However, moderate concentrations of melatonin (T3, T4) and high concentrations of melatonin (T5) may have toxic effects on cucumber seedlings. As the concentration increased, the root length decreased sharply by 32.27%, 37.82%, and 49.58%, respectively. In terms of stem diameter, the T0 group showed a 43.3% decrease compared to the CK group, indicating that salt stress may inhibit stem thickening by interfering with cell division or cell wall expansion, possibly due to reduced water absorption and ion toxicity caused by osmotic stress. Compared to T0, the stem diameters of T1–T4 groups significantly increased by 42.94%, 64.71%, 37.06%, and 37.06%, respectively, with T2 significantly higher than other melatonin treatment groups, indicating that low to moderate concentrations of melatonin (such as 50 μM) can effectively alleviate the inhibitory effect of salt stress on stem diameter. The stem diameter of the T5 treatment group was only 1.83, with no significant difference from the T0 group, indicating that high concentrations of melatonin may lose their mitigating effect on cucumber seedlings under salt stress or produce side effects. In terms of leaf area, the T0 leaf area was significantly lower than that of the control group, with a decrease of 47.25%, indicating that salt stress significantly inhibited the expansion of the cucumber seedling leaf area. This may be related to the decrease in photosynthetic capacity, accumulation of reactive oxygen species, and damage to membrane systems caused by salt stress. Compared with the T0 and CK groups, the T2 leaf area increased significantly (*p* < 0.05) by 13.54% and 115.26%, respectively, indicating that appropriate concentrations of melatonin not only alleviate salt stress inhibition but also promote leaf area growth. The leaf area of cucumber seedlings in the T1 and T3 treatment groups increased by 31.65% and 22.89%, respectively, which was higher than that of the salt stress group but significantly lower than that of the T2 group, indicating that the effect of melatonin is weakened when the concentration is insufficient or too high. The leaf area of the T4 and T5 treatment groups showed no significant difference from that of the T0 group, and in fact, it was even lower, indicating that high concentrations of melatonin may exacerbate stress damage. In terms of fresh and dry weights, compared with the control group (CK), the T0 group significantly reduced the fresh and dry weights of cucumber seedlings, with reductions of 56.67% and 63.27%, respectively, indicating that salt stress severely inhibited the accumulation of seedling biomass, which may be related to photosynthesis. The fresh weight of cucumber seedling leaves in the T2 group recovered to 76.9% of the CK, significantly higher than that of other treatment groups, indicating that this concentration effectively alleviated salt stress in cucumber seedlings. The T1 and T3 groups partially improved salt stress in cucumber seedlings. The fresh and dry weights of the T4 group showed no significant change compared to the T0 group. The fresh and dry weights of the T5 group were even lower than those of the T0 group, with decreases of 24.36% and 25.00%, respectively. This indicates that high concentrations of T5 may disrupt the redox balance and interfere with energy metabolism, thereby exacerbating stress-induced damage. This study demonstrates that the optimal melatonin (MT) concentration for alleviating salt stress differs between cucumber seeds and cucumber seedlings. The concentration of 50 μM MT (T2) was identified as the most effective treatment for mitigating salt stress in cucumber seedlings. However, further investigation is required to elucidate its underlying mechanism of action, which should involve the analysis of physiological indicators such as malondialdehyde (MDA) and superoxide dismutase (SOD) activity.

### 3.3. The Effects of MT Treatments at Different Concentrations on SOD, POD, CAT, and MDA in Cucumber Seedling Leaves and Roots Under Salt Stress

**Figure 3:**
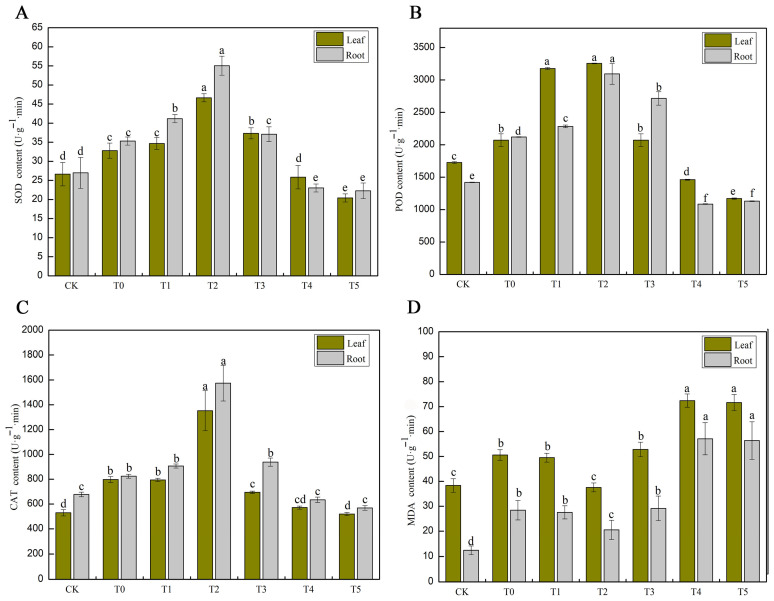
Melatonin application alleviates salt stress by scavenging ROS accumulation in leaves and roots of cucumber seedlings under salt stress. (**A**) Shows the contents of superoxide (SOD). (**B**) Shows the contents of peroxidase (POD). (**C**) Shows the contents of catalase (CAT). (**D**) Shows the contents of Malondialdehyde (MDA). Note: Values are means ± SD (*n* = 3); different letters indicate significant differences (*p* < 0.05).

The effect of salt stress on SOD activity in cucumbers is shown in Figure 3A. SOD activity in the T0 treatment group of cucumber leaves was 23.2% higher than that of the control group, and the SOD activity in the T0 group of cucumber roots was 30.9% higher than that of the CK group. This indicates that salt stress induces the accumulation of reactive oxygen species (ROS), triggering the activation of the antioxidant system, which includes the activation of SOD enzymes. These enzymes remove superoxide anions (O_2_^−^) and thereby alleviate oxidative damage. The analysis of the alleviating effect of melatonin on salt stress in cucumbers found that compared with the T0 treatment group, T2 treatment group had the most significant effect on increasing SOD activity in cucumber leaves and roots (29.65% increase in leaves and 55.97% increase in roots), indicating that this concentration can efficiently activate the antioxidant system and reduce salt stress damage. Additionally, low concentrations (25–100 μM) of melatonin can also promote SOD activity and enhance ROS clearance ability. In comparison, high concentrations (>150 μM) of melatonin inhibit SOD activity (in the T4 and T5 treatment groups, even lower than in the CK), which may be related to the metabolic burden or redox imbalance caused by the accumulation of melatonin itself. Moreover, the increase in SOD activity in roots was generally higher than that in leaves (in the T2 treatment group: +55.97% in roots vs. +29.65% in leaves), as roots are directly exposed to salt ion stress, and melatonin may preferentially regulate the ion balance and antioxidant response in the root system. Therefore, low concentrations of melatonin enhance the antioxidant defense of cucumbers. In contrast, high concentrations of melatonin (>150 μM) have negative effects, which may interfere with cellular redox homeostasis, inhibit SOD synthesis, or trigger feedback regulation, leading to a decrease in enzyme activity. This finding is consistent with previous studies [33].

The effect of salt stress on POD activity in cucumbers is shown in Figure 3B. Salt stress significantly induced an increase in POD activity. The POD activity in the T0 group of cucumber leaves was 20.0% higher than that in the CK, and the POD activity in the T0 treatment group of roots was 49.3% higher than that in the CK. This indicates that salt stress activates the antioxidant system, and the response intensity in roots is higher than that in leaves, as roots are in direct contact with salt ions and require higher activity to remove reactive oxygen species such as hydrogen peroxide (H_2_O_2_). The analysis of the alleviating effect of melatonin on salt stress in cucumbers revealed that compared with the T0 treatment group, T2 treatment group was the optimal concentration, with the POD activity in leaves reaching its peak, increasing by 57.1% compared to the salt-stressed group, and the POD activity in roots also reaching its peak, increasing by 45.9% compared to the salt-stressed group. Additionally, low concentrations (25–50 μM) of melatonin significantly increased POD activity. The POD activity in leaves approached its peak in the T1 group, while in roots, it reached its maximum in the T2 treatment group. At medium–high concentrations (100 μM MT), the POD activity in leaves decreased to the salt-stressed level, but compared with the T0 treatment group, the POD activity in roots still maintained a 28.1% increase, indicating that roots have a stronger tolerance to melatonin. At high concentrations (≥150 μM), the POD activity was significantly inhibited (decrease in leaves/roots >29%), which may be related to the imbalance of redox or metabolic negative feedback caused by excessive melatonin.

The effect of salt stress on CAT activity in cucumber is shown in Figure 3C. Salt stress significantly induced an increase in CAT activity. The CAT activity in the T0 treatment group of cucumber leaves was 50.7% higher than that of CK, indicating that salt stress induced H_2_O_2_ accumulation and triggered an increase in CAT activity to remove excessive hydrogen peroxide. The CAT activity in the T0 treatment group of roots was 21.7% higher than that of CK, with a lower increase than that in leaves, possibly due to the fact that the root system preferentially activates other antioxidant pathways (such as APX/GR). The regulatory effect of melatonin on CAT activity under salt stress in cucumber showed that, compared with the respective T0 treatment group, CAT activity in leaves increased by 69.6% and in roots by 91.0%, significantly higher than in other treatment groups. Additionally, low concentrations (≤50 μM) of melatonin significantly promoted CAT activity in cucumber, with 50 μM MT being the peak point. Medium and high concentrations (T3) of melatonin caused the CAT activity in leaves to drop below the T0 level. At the same time, in roots, it still maintained a 13.8% increase, indicating that the root system has a stronger tolerance to high concentrations of MT. High concentrations (T4 and T5 treatment groups) of melatonin significantly inhibited CAT activity in cucumber (decrease in leaves/roots >23%), possibly due to an imbalance in reactive oxygen species homeostasis or feedback inhibition caused by excessive MT [34].

The effect of salt stress on MDA activity in cucumbers is shown in Figure 3D. The MDA content in the T0 treatment group of cucumber leaves increased by 31.9% compared to CK, indicating that salt stress induced intensified membrane lipid peroxidation and cell membrane damage. The MDA content in the roots of the T0 treatment group increased by 128.4% compared to the CK, a much higher increase than that in the leaves, suggesting that the roots are more sensitive to salt stress and suffer more severe membrane damage. The regulatory effect of melatonin on MDA accumulation demonstrated that, compared with the T0 group, application of 50 μM MT (T2) significantly reduced membrane lipid peroxidation, resulting in a 25.7% decrease in MDA content in leaves and a 27.7% decrease in roots. Additionally, the MDA content in cucumbers treated with low concentrations (T2) of melatonin was significantly lower than that in the T0 group and even approached the CK level; at medium and high concentrations (T3), the MDA content in leaves exceeded that of the T0 group (+4.4%), while in roots it only slightly increased (+2.6%), indicating that leaves are more sensitive to high concentrations. At high concentrations (T4 and T5 treatment groups), the MDA content in cucumbers increased by 43% to 101% compared to the T0 group, and melatonin turned into a pro-oxidant, exacerbating membrane damage.

### 3.4. The Effect of Different Concentrations of MT on Soluble Protein in Cucumber Seedling Leaves and Roots Under Salt Stress

**Figure 4:**
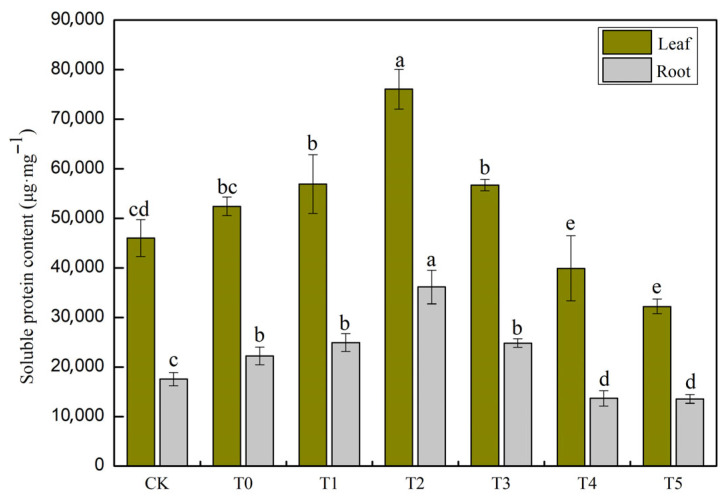
Effects of exogenous MT on the content of soluble protein in leaves and roots of cucumber seedlings under salt stress. Note: Values are means ± SD, *n* = 3; different letters indicate significant differences (*p* < 0.05).

The effects of salt stress on soluble protein content in cucumbers are presented in Figure 4. In the T0 treatment group, the soluble protein content in cucumber leaves was 14.0% higher than in the CK, indicating that salt stress induces the synthesis of osmoprotective substances to maintain cellular homeostasis. In roots, the T0 treatment group exhibited a 26.6% increase in soluble protein content compared to CK, a greater increase than that observed in leaves. This suggests that roots, as the primary site of stress exposure, require stronger osmotic regulation. The results of the regulatory effect of melatonin on soluble protein content demonstrated that 50 μM melatonin (T2 treatment group) significantly enhanced soluble protein levels in salt-stressed plants. In leaves, the increase reached 45.0% compared to T0 (*p* < 0.05), representing the maximum observed effect; in roots, the increase was 62.6% compared to T0 (*p* < 0.05). At 100 μM melatonin (T3 treatment group), soluble protein content in both leaves and roots declined to levels comparable to T1 (8–12% increase), which was significantly lower than T2 (*p* < 0.05). Melatonin concentrations of 150 μM or higher imposed stress on cucumbers and exhibited inhibitory effects. In T4 and T5 treatment groups, the soluble protein content in leaves decreased by 24.0% and 38.6%, respectively, compared to T0 treatment group In roots, the decreases were 38.6% and 39.1%, respectively. In summary, by significantly increasing soluble protein content (leaves: +45.0%, roots: +62.6%) and synergistically enhancing osmotic regulation and antioxidant capacity, 50 μM melatonin represents the optimal concentration for mitigating salt stress in cucumbers.

### 3.5. The Impact of Different Concentrations of MT on Chlorophyll Content in Cucumber Seedling Leaves and Roots Under Salt Stress

**Figure 5:**
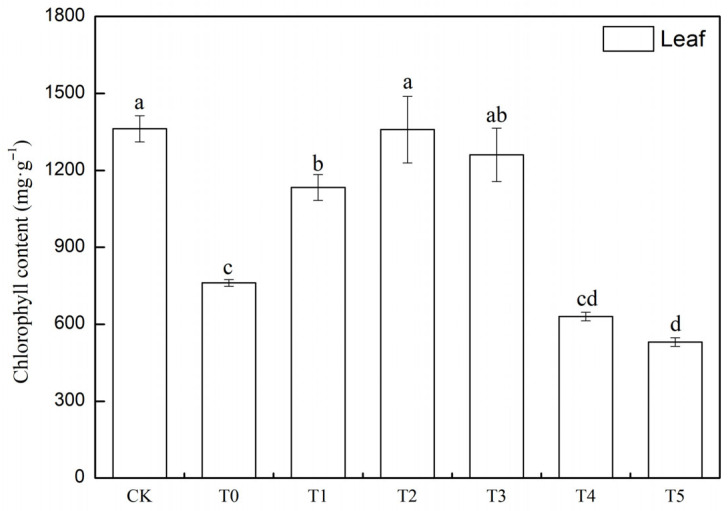
Effects of Exogenous MT (Melatonin) on Chlorophyll Content in Leaves of Cucumber Seedlings under Salt Stress. Note: Values are means ± SD (*n* = 3); different letters indicate significant differences (*p* < 0.05).

Based on the analysis of Figure 5, it is found that the chlorophyll content in the leaves of cucumber seedlings in the T0 treatment group is significantly lower than that in CK, with a decrease of 44.1%, indicating that salt stress severely damages photosynthetic capacity by disrupting the photosynthetic membrane system. Compared with the CK, the chlorophyll content in the leaves of the T1 treatment group recovers to 83.2% of the control, and the chlorophyll content in the T2 treatment group and the medium-concentration T3 treatment group are significantly lower than that in T2 but still higher than that in T0, indicating that the protective effect of medium-concentration melatonin gradually weakens. The chlorophyll content in the leaves of cucumber seedlings in the high-concentration melatonin groups T4 and T5 treatment groups is even lower than that in the salt stress group, indicating that high-concentration melatonin induces metabolic disorders. Based on the experimental data, melatonin exhibits a bidirectional regulatory characteristic of “promoting at low concentrations and inhibiting at high concentrations” on the loss of chlorophyll in cucumber seedling leaves induced by salt stress. T2 treatment group is the optimal treatment concentration, which fully restores chlorophyll levels through the synergistic action of antioxidant defense and photosynthetic repair. In contrast, T5 (high concentration) may exacerbate damage by interfering with ion homeostasis or inducing secondary oxidative stress.

### 3.6. Correlation Analysis, Principal Component Analysis, and Comprehensive Evaluation Using the Membership Function of Exogenous Melatonin Treatment on Cucumber Seedlings Under Salt Stress

**Figure 6:**
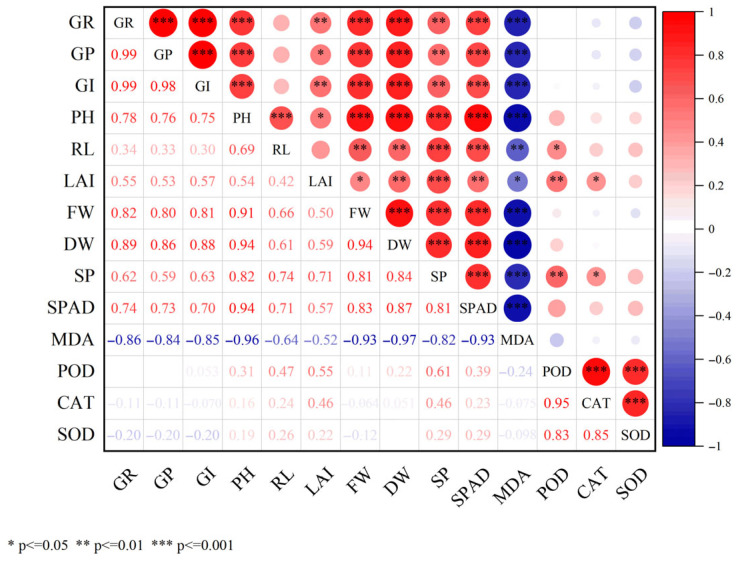
Correlation analysis matrix for each indicator. Note: GR stands for germination rate; GP for germination potential; GI for germination index; PH for plant height; RL for root length; LAI for leaf area index; FW for fresh weight; DW for dry weight; SP for soluble protein; SPAD for relative chlorophyll content; MDA for malondialdehyde; POD for peroxidase; CAT for catalase; SOD for superoxide dismutase. * *p* ≤ 0.05, ** *p* ≤ 0.01, *** *p* ≤ 0.001. Red tones indicate positive correlations (positive correlation coefficients ranging from light red to dark red, corresponding to correlation coefficients from 0 to 1, with darker shades representing stronger positive correlations). Blue tones indicate negative correlations (negative correlation coefficients ranging from light blue to dark blue, corresponding to correlation coefficients from 0 to −1, with darker shades representing stronger negative correlations). White represents the absence of a statistically significant correlation (correlation coefficient close to 0 or non-significant correlation).

The correlation analysis of each index is shown in Figure 6. There is a highly significant positive correlation among all the indices. The correlation coefficients between GI and GR, as well as GP, are very large, at 0.99 and 0.98, respectively. The correlation coefficients between PH and FW, DW, and SPAD are relatively large, being 0.91, 0.94, and 0.94, respectively. The correlation coefficient between FW and DW is relatively large, being 0.94. All the indices except MDA and some (POD, CAT, and SOD) are positively correlated; MDA and some (POD, CAT, and SOD) are negatively correlated with other indices. This indicates that the changes in these indices are mostly consistent, and there is an overlap among them. Therefore, multivariate statistical methods should be adopted to further analyze all the indices. Additionally, there are three pairs of strong positive correlations, such as the representative index pair DW-MDA (correlation coefficient is 0.97), indicating a negative feedback between dry weight and damage. The index pair CAT-POD (correlation coefficient is 0.95) indicates the synergistic effect of antioxidant enzymes. The index pair FW-PH (correlation coefficient is 0.91) indicates the association between biomass and morphology. There are two strong negative correlations, such as the index pair MDA-SPAD (correlation coefficient is −0.93), indicating that damage inhibits photosynthesis. The index pair MDA-GR (correlation coefficient is −0.86) indicates that damage inhibits germination.

**Figure 7:**
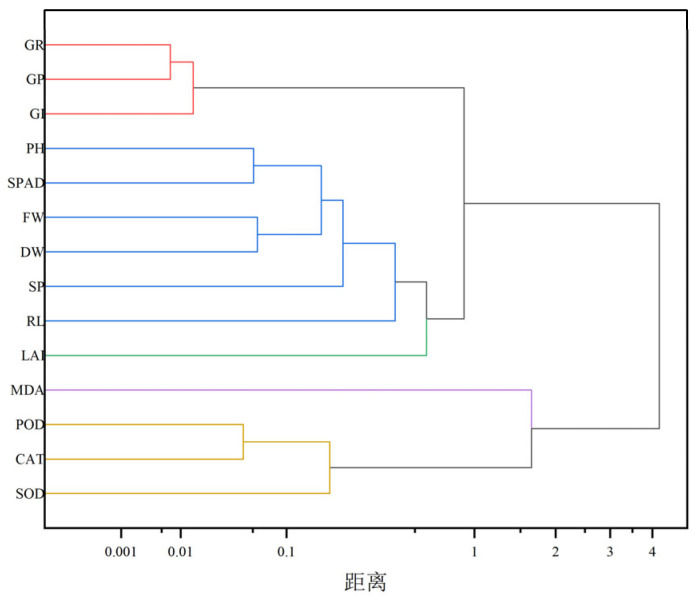
Cluster Analysis Matrix of Each Indicator. Note: GR stands for germination rate; GP for germination potential; GI for germination index; plant height for PH; root length for RL; leaf area index for LAI; fresh weight for FW; dry weight for DW; soluble protein for SP; relative chlorophyll content SPAD; malondialdehyde for MDA; peroxidase for POD; catalase for CAT; superoxide dismutase for SOD. The different colors (red, blue, green, purple, and yellow) represent distinct clusters in the figure. Variables sharing the same color are grouped within the same cluster, indicating a high degree of similarity in their data features, as reflected by shorter distances and stronger associations.

**Figure 8:**
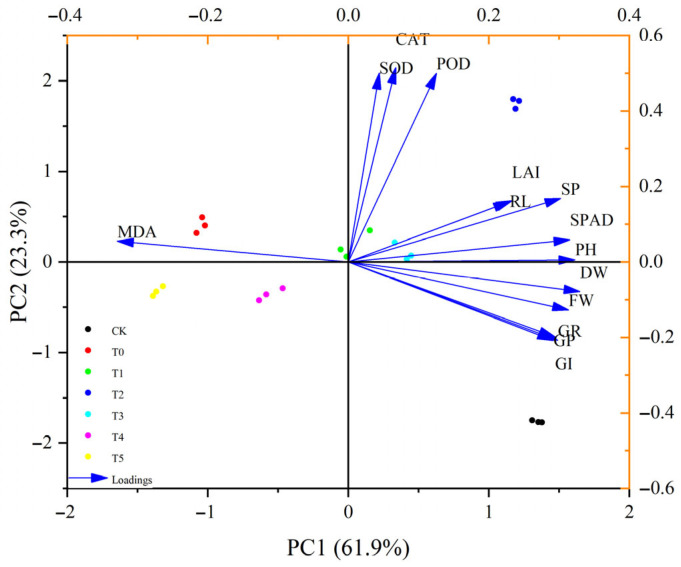
Principal component load score analysis. PC1 and PC2 represent the primary dimensions reflecting sample distribution characteristics. The differently colored points correspond to samples from distinct experimental groups in the figure, providing a visual representation of inter-group differences and clustering patterns within the principal component space. Black (CK) denotes the control group (Control group), serving as a baseline for comparison with other experimental groups to illustrate sample characteristics in the absence of specific treatments. Red (T0), Green (T1), Blue (T2), Cyan (T3), Purple (T4), and Yellow (T5) represent experimental groups T0, T1, T2, T3, T4, and T5, respectively.

Cluster analysis of exogenous melatonin treatment on cucumber seedlings under salt stress. The results of this study (Figure 7) show that the 14 indicators were classified into 5 clusters by systematic clustering. The first cluster (germination indicators): GR-GP-GI were closely clustered (distance < 0.1), indicating that germination rate, germination potential, and germination index responded highly synergistically to salt stress. The second cluster (growth indicators): PH-RL-DW-FW were grouped together, demonstrating the intrinsic connection between morphology and biomass; PH and RL were co-varying (r = 0.69, *p* < 0.001); root and shoot grew in coordination with a strong link between DW and FW (r = 0.94); and dry matter accumulation was dependent on fresh weight. The third cluster (damage markers): MDA was independently clustered (distance from other clusters > 1.2), indicating a unique response pattern of oxidative damage. The fourth cluster (antioxidant enzyme system): POD-CAT-SOD were super closely clustered (distance < 0.05); POD and CAT were nearly coincident (r = 0.95, *p* < 0.001); and SOD was highly correlated with POD/CAT (r = 0.83–0.85). The fifth cluster (photosynthetic indicators): SPAD-LAI were moderately associated (distance 0.5), reflecting the functional complementarity between chlorophyll content and leaf area. The overall regulation pattern of melatonin is hierarchical, with the priority of activating antioxidant enzymes (POD/CAT/SOD↑)→ alleviating oxidative damage (MDA↓)→ promoting photosynthesis (SPAD↑)→ driving biomass accumulation (PH/RL/FW/DW↑). As can be seen from Figure 8, the horizontal axis PC1 (60.9%) represents the growth-stress balance axis, with the positive region (on the right) correlating with growth recovery indicators (GR, PH, and SPAD) and the negative region (on the left) correlating with oxidative damage indicators (MDA, CAT). The vertical axis PC2 (21.4%) represents the antioxidant response axis, with the positive region (at the top) correlating with high antioxidant enzyme activity (POD, SOD) and the negative region (at the bottom) correlating with inhibition of the antioxidant system. The dominant contribution of PC1 (60.9%) indicates that physiological changes in plants under salt stress are primarily driven by the antagonistic relationship between biomass accumulation capacity and membrane damage severity. PC2, on the other hand, reveals the independent regulatory mechanism of the antioxidant enzyme system.

Through principal component analysis of the following 14 indicators, the overall impact of different indicators on cucumber seedlings was comprehensively evaluated. As shown in Table 3, the maximum eigenvalue was 8.66292, the maximum contribution rate was 61.88%, and the cumulative contribution rate of the first three principal components was 91.94%, exceeding 85.000%. Therefore, the first three components can be extracted as the indicators for the subsequent membership function analysis.

The PCA biplot analysis of principal component analysis shows in Figure 8 that the horizontal axis PC1 contributes 61.9% (dominant variation), and the vertical axis PC2 contributes 23.3% (auxiliary variation), with a cumulative explanatory rate of 85.2%, indicating that the two principal components can fully reflect the data variation. From the distribution pattern of the treatment groups, it is found that the T0 group (salt stress group) is concentrated in the negative direction of PC1 (in the same direction as the MDA arrow), indicating a high damage state. The T2/T3 groups (melatonin treatment) are located in the positive direction of PC1 (in the same direction as the PH/FW/DW arrows), indicating the optimal biomass. The CK is close to the origin, representing the basic physiological state.

The PCA analysis of the comprehensive evaluation of the membership function (Table 4) indicated that PC1 (growth-stress balance axis) and PC2 (antioxidant axis) jointly explained 82.31% of the physiological variation. In the melatonin treatment groups, the 50 μM concentration (T2) had a significantly higher PC1 score (1.0722) compared to the salt stress group (T0: −0.9365, *p* < 0.01), and its PC2 score (0.3544) indicated a strong antioxidant response. The comprehensive evaluation of the membership function confirmed that the T2 group had the highest D value (0.7155, Table 4), suggesting that this concentration effectively alleviated salt stress by synergistically promoting growth indicators (PH, DW) and antioxidant enzyme activities (SOD, POD). The comprehensive evaluation concluded that: T2 > T1 > CK > T5 > T3 > T4 > T0.

### 3.7. Melatonin Regulates the Salt Stress-Responsive Genes

**Figure 9:**
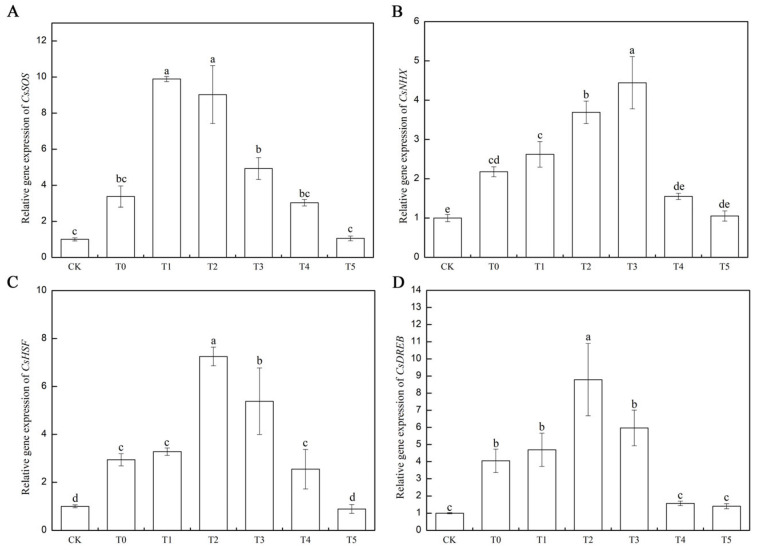
Melatonin alleviates drought stress by regulating salt stress response genes. (**A**,**B**) show the relative expression of *CsSOS* and *CsNHX* salt stress response genes, while (**C**,**D**) show the relative expression levels of *CsHSF* and *CsDREB* drought response genes in cucumber. Note: Data were analyzed in three independent biological replicates (± standard deviation, SD), and different lowercase letters indicate significant differences among treatments (*p* < 0.05). CK: Distilled water as a control, T0: 150 mM salt treatment; T1: S + 25 μM MT group; T2: S + 50 μM MT group; T3: S + 100 μM MT group; T4: S + 150 μM MT group; T5: S + 200 μM MT group; S: 150 mM salt treatment.

The combined effect of salt stress and melatonin-added salt stress significantly influenced the expression of these stress-related genes. Compared to the control plants (CK), the gene expression of *CsSOS* (Cucsa.026490) increased by 237% 48 h after salt stress application (T0). Compared to T0 under salt stress alone, the gene expression of *CsSOS* under melatonin and salt combinations (T1, T2, T3, and T4) increased to 196.30%, 167.84%, and 46.19%, respectively. However, the gene expression of *CsSOS* under T4 and T5 combinations decreased to 10.10% and 68.80%, respectively (Figure 2 and Figure 9A). Similarly, compared to the control (CK) plants, the expression of *CsNHX* (Cucsa.363260) significantly increased by 117.69% under salt stress (T0) 48 h after stress application. Compared to T0 under salt stress alone, the gene expression of *CsNHX* under melatonin and salt combinations (T1, T2, T3, and T4) increased to 20.43%, 69.54%, and 104.12%, respectively. However, the gene expression of *CsNHX* under T4 and T5 combinations decreased to 28.76% and 31.58%, respectively (Figure 2 and Figure 9B). Additionally, 48 h after salt stress application, the gene expression of *CsHSF* (Cucsa.321980) under salt stress (T0) increased by 193.90% compared to the control plants (CK). Compared to T0 under salt stress alone, the gene expression of *CsHSF* under melatonin and salt combinations (T2, T3) increased to 146.26% and 82.99%, respectively. There were no significant differences in the gene expression of *CsHSF* between T1 and T4 compared to T0 under salt stress alone. Similarly, there were no significant differences in the gene expression of *CsHSF* between T5 and CK (Figure 2 and Figure 9C). Similarly, 48 h after salt stress application, the gene expression of *CsDREB* (Cucsa.136780) under salt stress (T0) increased by 304.94% compared to the control plants (CK); Compared to T0 with salt stress alone, there was no significant difference in the T1 combination. However, under the melatonin and salt combinations (T2, T3, T4), the gene expression of *CsHSF* increased to 117.33% and 47.52%, respectively. Meanwhile, under the T4 and T5 combinations, the gene expression of *CsDREB* decreased to 57.00% and 65.35%, respectively (Figure 2 and Figure 9D).

## 4. Discussion

Salt stress significantly inhibits plant growth and development [35]. Under salt stress, chlorophyll accumulation in wheat leaves is suppressed, and both root length and plant height are markedly reduced [36,37,38]. Studies have demonstrated that salt stress significantly decreases plant height, leaf area, and biomass in cotton seedlings, whereas exogenous melatonin (MT) treatment can significantly mitigate these adverse effects [16,39]. Our findings indicate that salt stress severely inhibits cucumber seed germination and seedling growth. However, the MT application significantly restores the phenotypic traits and growth parameters of cucumber seeds under salt stress. Among the tested concentrations, 100 μM MT exhibited the most pronounced effect in alleviating the inhibitory impact of salt stress on seed germination (Figure 1 and Table 1). Furthermore, MT treatment significantly increased plant height, stem diameter, leaf area, fresh weight, and dry weight in salt-stressed cucumber seedlings. However, these parameters remained lower than those of the control group. Further analysis revealed that 50 μM MT (T2) was most effective in alleviating salt-induced growth inhibition in seedlings (Figure 2 and Table 2). These results suggest that appropriate concentrations of exogenous melatonin can effectively mitigate the negative effects of salt stress on cucumber seedling growth. In contrast, high MT concentrations may weaken the ameliorative effect or even exacerbate stress inhibition. Similar observations have been reported in seedlings of Lepidium apetalum and rice under salt stress [40,41]. This phenomenon may be attributed to the role of melatonin in promoting root development [42] and assisting plants in maintaining water balance [43], thereby alleviating salt-induced water stress and enhancing the cucumber’s capacity to absorb water and nutrients. Moreover, melatonin exhibits a concentration-dependent differential effect in alleviating salt stress-induced inhibition in cucumber seeds and seedlings (100 μM being more effective for seeds, and 50 μM more effective for seedlings). This difference may be due to the fact that seed germination requires a higher melatonin concentration to overcome physical barriers and activate relevant signaling pathways. In contrast, seedlings exhibit a more sensitive physiological response to melatonin, with lower concentrations being sufficient to regulate growth-related gene expression and metabolic processes.

Chlorophyll, as a critical photosynthetic pigment, plays a fundamental role in plant physiological processes by absorbing and transferring light energy. Increased chlorophyll content contributes to enhanced photosynthetic efficiency in plants [44]. Hareem et al. [45] reported that with increasing salt concentration, the levels of chlorophyll a, chlorophyll b, and total chlorophyll in Fenugreek exhibited a progressive decline. The results of this study demonstrate that under salt stress conditions, the application of 50 μM melatonin (T2) significantly elevates chloroplast pigment content in cucumber seedlings (Figure 5). In contrast, the promoting effects of 100, 150, and 200 μM MT on chloroplast pigment synthesis gradually diminish with increasing concentration, aligning with previous studies. The observed effect may be attributed to melatonin’s ability to enhance the efficiency of the chloroplast ascorbate-glutathione cycle and accelerate the scavenging of reactive oxygen species, thereby mitigating salt stress-induced damage to the chloroplasts [46]. Additionally, it may be closely associated with melatonin’s inhibitory effect on chlorophyll-degrading enzyme activity [47]. Chlorophyll-degrading enzymes play a crucial role in the breakdown of chlorophyll; when their activity is suppressed, chlorophyll degradation slows, resulting in its prolonged retention in plant tissues. Elevated chlorophyll content reflects an improved capacity for light absorption and energy conversion, which enhances photosynthetic performance and consequently improves plant tolerance to salt stress.

The antioxidant system plays a vital role in plant life cycles by protecting plants against adverse environmental conditions [48]. Under normal physiological conditions, the production and scavenging of reactive oxygen species (ROS) maintain a dynamic equilibrium. However, high-salt stress disrupts this balance, leading to the accumulation of superoxide anions and malondialdehyde (MDA), which negatively affect plant growth and development [49]. Under stress conditions, plants typically enhance the activities of antioxidant enzymes such as superoxide dismutase (SOD), peroxidase (POD), and catalase (CAT) to eliminate excessive ROS and mitigate oxidative damage [50]. Melatonin can protect cells from oxidative stress by enhancing the activity of intracellular antioxidant enzymes and directly scavenging ROS [51]. Li’s [52] study demonstrated that melatonin significantly increases antioxidant enzyme activity in cotton seeds and reduces oxidative damage, which may be attributed to the upregulation of antioxidant enzyme-related genes by exogenous melatonin and the reduction in biological macromolecule degradation, thereby enhancing enzymatic activity [53]. The findings of this study are consistent with previous reports (Figure 3), indicating that an appropriate concentration of melatonin can activate SOD, POD, and CAT in both leaves and roots of cucumber seedlings under salt stress while simultaneously reducing MDA levels. Among the tested concentrations, 50 μM MT (T2) most effectively enhances the antioxidant defense system, thereby alleviating oxidative damage. Furthermore, the increase in CAT activity in roots is significantly greater than in leaves, likely due to the direct exposure of roots to ionic stress, where melatonin preferentially enhances H_2_O_2_ scavenging capacity. The POD activity of leaves reaches its peak at 25 μM MT (T1), whereas the POD of roots requires 50 μM MT (T2), suggesting that leaves are more sensitive to low melatonin concentrations. However, when high concentrations of exogenous melatonin are applied, a pro-oxidant effect is observed, characterized by a simultaneous decline in SOD, POD, and CAT activities and a sharp increase in MDA content, indicating a shift in melatonin from an antioxidant to a pro-oxidant. The exact mechanism underlying this pro-oxidant effect remains unclear. It is yet to be determined whether melatonin exerts its effects through direct interaction with existing enzymes or via a signaling pathway that regulates gene expression to promote enzyme synthesis.

Studies have demonstrated that salt stress can elevate ion concentrations within plants, potentially disrupting normal physiological and metabolic processes [54]. To adapt to saline environments, plants typically accumulate osmotic adjustment substances, which reduce cellular osmotic potential and the freezing point, thereby enhancing their capacity to cope with environmental fluctuations [55]. Previous studies have shown that under low-salt conditions, the content of soluble proteins in plants initially increases; however, prolonged stress leads to a decline in the capacity for osmotic adjustment [16,56]. Under salt stress, soluble protein levels decrease in naked oats, whereas proline content significantly rises; Exogenous melatonin (MT) treatment has been found to markedly enhance the accumulation of both soluble proteins and proline [57]. Our experimental results indicate that the appropriate application of exogenous melatonin under salt stress can effectively increase soluble protein content in both cucumber seedlings and their roots (Figure 4). Notably, 50 μM MT (T2) treatment significantly enhances cellular osmotic adjustment capacity, thereby improving cucumber salt tolerance. However, when MT concentration reaches or exceeds 100 μM (T3), its ameliorative effect diminishes and may even exhibit inhibitory effects. This may be due to high MT concentrations suppressing antioxidant enzyme activity and protein synthesis, leading to the accumulation of reactive oxygen species (ROS) and damage to the membrane system, thereby creating a negative feedback loop. These findings align with previous reports, suggesting that melatonin alleviates salt-induced osmotic imbalance by promoting the accumulation of inorganic ions and organic solutes, thereby increasing cell sap concentration, lowering osmotic potential, and enhancing cellular water retention, which improves plant salt tolerance [22]. Additionally, emerging evidence indicates that melatonin aids in maintaining ion homeostasis and mitigating salt-induced ion imbalance [58]. The application of exogenous melatonin can effectively enhance osmotic adjustment capacity under salt stress, promote the accumulation of osmoprotectants, reduce oxidative damage, and thus support normal seedling growth [59].

Analysis of the hierarchical characteristics of the melatonin regulatory network revealed that cluster analysis and principal component analysis collectively identified a three-tiered response pattern of melatonin in mitigating salt stress (Figure 6, Figure 7 and Figure 8). The primary response was characterized by the preferential activation of the antioxidant enzyme system in the presence of 50 μM MT (T2), with a strong correlation among POD, CAT, and SOD (r > 0.83). This activation led to a reduction in membrane lipid peroxidation through the scavenging of reactive oxygen species (ROS), as indicated by the formation of an independent cluster of MDA, with a distance greater than 1.2 from the antioxidant enzyme system. The secondary response was characterized by a significant improvement in photosynthetic parameters (SPAD-LAI clustering), with principal component 2 (PC2) accounting for 23.3% of the overall variance. During this phase, chlorophyll content and PSII efficiency gradually recovered. The tertiary response involved the coordinated enhancement of biomass accumulation (PH-RL-DW-FW clustering, r = 0.94) and germination indices (GR-GP-GI clustering, r = 0.98), with principal component 1 (PC1) accounting for 61.9% of the total variance. This response pattern aligns with the “first antioxidation, then growth promotion” mechanism of melatonin observed in alfalfa; however, cucumber exhibited greater sensitivity to MT concentration, with 50 μM MT achieving optimal effects [60]. Membership function analysis demonstrated that 50 μM MT (T2) had the highest comprehensive evaluation value D (0.7155), reflecting advantages in three key areas: dual-system synergy, metabolic balance, and organ coordination. First, both PC1 (growth axis) and PC2 (antioxidant axis) scores were significantly higher than those of the control group T0 (*p* < 0.01), indicating simultaneous enhancement of antioxidant defense and carbon assimilation. Second, in terms of metabolic balance, 50 μM MT avoided the potential feedback inhibition associated with high-concentration treatments (T4/T5). Third, with respect to organ coordination, the increase in SOD activity in roots was significantly greater than in leaves, suggesting that melatonin preferentially protects absorptive organs to maintain overall plant stability. Using multivariate statistical methods, this study confirmed that cucumber exhibits organ-specific thresholds in response to salt stress, with roots showing higher tolerance concentrations than leaves—a pattern distinctly different from the uniform systemic response observed in alfalfa.

Furthermore, this study investigated the effects of varying melatonin concentrations on the expression levels of *CsSOS*, *CsNHX*, *CsHSF*, and *CsDREB* genes in cucumber seedlings under salt stress. These genes have been extensively studied and are widely recognized as key regulatory factors involved in cucumber’s response to salinity. Previous research has demonstrated that different levels of salt stress can significantly influence the expression of stress-related genes in rice. Further research has demonstrated that overexpression of the *CsSHMT3* gene in cucumber seedlings significantly up-regulates the expression levels of stress-responsive genes, including *SOD*, *CAT*, *SOS1*, *SOS2*, *NHX*, and *HKT*, thereby enhancing the salt tolerance of cucumber seedlings. In contrast, under salt stress conditions, silencing of the *CsSHMT3* gene leads to a significant down-regulation of these stress-related genes, consequently reducing salt tolerance [61]. During the early phase of salt stress, salt-tolerant plants exhibit rapid up-regulation of *OsSOS3* and *OsNHX1* expression, representing a short-term response, while *OsHKT* is progressively up-regulated and *OsSOS1* is down-regulated throughout the entire stress duration. In contrast, salt-sensitive plants display a diminished induction of *OsSOS3*, *OsNHX1*, and *OsHKT* at the initial stage of stress, reflecting a reduced capacity for stress response [62]. Members of the *OsNHX* gene family exhibit significant regulatory responses under salt stress conditions, with *OsNHX1* overexpression notably improving salt tolerance in transgenic rice [63]. Theerawitaya et al. [21] reported that under 200 mM NaCl stress, the expression level of *OsNHX1* was elevated in the leaves of rice cultivars Pokkali and IR29. Similarly, this study observed a significant upregulation of *CsSOS* and *CsNHX* gene expression in cucumber under salt stress conditions (Figure 9A–C). Previous studies have demonstrated that exogenous melatonin enhances plant salt tolerance through the up-regulation of genes associated with the SOS signaling pathway in wheat, particularly by significantly increasing the expression levels of SOS1, SOS2, and SOS3 under salt stress conditions [64]. Additionally, melatonin treatment has been shown to induce the overexpression of ion transporters such as *NHX1-4* in tomatoes under NaCl stress [65]. Consistent with previous findings, this study confirmed that low concentrations of melatonin (T1–T3) effectively mitigated the adverse effects of salt stress on cucumber seedlings by modulating the expression of *CsSOS* and *CsNHX* genes (Figure 9A,B). Research has also shown that melatonin pretreatment promotes the transcriptional activation of *OsSOS* and *OsNHX* genes in rice under salt stress, thereby facilitating Na+ exclusion and sustaining plant stress resistance [22].

Studies have demonstrated that multiple transcription factors have been identified, which play crucial regulatory roles in plant responses to various abiotic stresses [22,65]. In the rice cultivar AP2, *OsDREB1A* exhibits significant upregulation under drought conditions [66]. Previous studies have reported that the miR165/166-PHABULOSA module promotes thermotolerance by transcriptionally and post-transcriptionally regulating HSFA1 in Arabidopsis [67]. Recent findings indicate that the overexpression of *AeHSFA2b* in Arabidopsis significantly improves tolerance to salt stress by increasing the expression of AtRS5, AtGolS1, and AtGolS2 [68]. Furthermore, Samtani et al. have confirmed that *HSFA3* participates in the signaling pathway of drought stress [23]. The results of this study show that, under single-salt-stress conditions, the expression levels of *CsHSF* and *CsDREB* genes in cucumber are markedly upregulated (Figure 9C,D), suggesting that these genes play important regulatory roles in cucumber’s response to salt stress. In recent years, substantial progress has been made in elucidating the specific roles of melatonin in plant responses to abiotic stress. Research indicated that melatonin pretreatment enhances plant tolerance to various abiotic stresses, including low temperature, high temperature, salinity, and drought, through the regulation of key gene expressions in the DREB/CBF, HSF, SOS, and ABA signaling pathways, respectively [69]. This study found that under salt stress, low-concentration melatonin treatment significantly increased the expression levels of *CsHSF* and *CsCREB* genes in cucumber leaves (Figure 9C,D), with the T2 treatment showing the most pronounced effect. In contrast, high-concentration melatonin treatment did not result in significant differences in *CsHSF* and *CsCREB* expression compared to salt stress alone.

## 5. Conclusions

In summary, we hypothesize that low concentrations of melatonin can enhance the cucumber’s response to salt stress. Salt stress significantly inhibits the germination efficiency of cucumber seeds and hinders the normal growth of seedlings. Under salt stress conditions, the application of 50 μM exogenous melatonin can effectively promote the morphological development of cucumber seedlings, specifically by significantly increasing the length and thickness of the hypocotyls and roots while enhancing the biomass accumulation of the plants. From a physiological mechanism perspective, this concentration of melatonin significantly improves the salt tolerance of seedlings by maintaining cell osmotic balance, enhancing membrane system stability, and scavenging reactive oxygen species (ROS). Additionally, melatonin treatment can promote chlorophyll synthesis in leaves and activate the antioxidant enzyme system, thereby enhancing the photosynthetic performance and stress resistance of seedlings. The effect of melatonin on plants exhibits a typical “low-promoting and high-suppressing” effect, with higher concentrations conversely exacerbating salt damage. From a gene regulation perspective, melatonin appears to induce or activate the expression of specific resistance genes, thereby enhancing plant tolerance to salt and drought stress. Further research is needed to fully understand the mechanisms and signaling pathways involved in melatonin’s response to cucumber salt stress. This study provides new insights for improving crop cultivation techniques in arid and semi-arid regions and has important practical value for addressing environmental stresses such as soil salinization.

## Figures and Tables

**Table 1:** Effects of Exogenous MT (Melatonin) on Germination and Growth of Cucumber Seeds under Salt Stress.

Treament	Hypocotyl Length/mm	Root Length/mm	Thick Hypocotyl/mm	Diameter of Root/mm	Fresh Weight/g	Dry Weight/g
CK	83.83 ± 1.59 ^a^	102.10 ± 0.11 ^a^	2.13 ± 0.02 ^a^	1.70 ± 0.01 ^a^	1.68 ± 0.25 ^a^	0.08 ± 0.01 ^a^
T0	16.1 ± 4.2 ^d^	25.43 ± 0.09 ^e^	1.33 ± 0.01 ^c^	1.33 ± 0.01 ^b^	0.42 ± 0.1 ^c^	0.06 ± 0 ^c^
T1	53.77 ± 4.05 ^c^	48.70 ± 0.34 ^d^	1.63 ± 0.01 ^b^	1.47 ± 0.01 ^b^	0.95 ± 0.12 ^b^	0.06 ± 0 ^c^
T2	65.6 ± 2.9 ^b^	54.77 ± 0.33 ^c^	1.70 ± 0.01 ^b^	1.30 ± 0.02 ^b^	1.06 ± 0.09 ^b^	0.07 ± 0.01 ^bc^
T3	65.1 ± 3.04 ^b^	68.20 ± 0.34 ^b^	2.00 ± 0.02 ^a^	1.90 ± 0.01 ^a^	1.81 ± 0.15 ^a^	0.08 ± 0.01 ^ab^
T4	77.5 ± 2.72 ^a^	49.10 ± 0.39 ^cd^	1.70 ± 0.01 ^b^	1.03 ± 0.01 ^c^	1.05 ± 0.04 ^b^	0.07 ± 0 ^c^
T5	55.37 ± 3.52 ^c^	45.67 ± 0.33 ^d^	1.50 ± 0.01 ^bc^	1.30 ± 0.01 ^b^	0.82 ± 0.14 ^b^	0.06 ± 0 ^c^

Note: The values represent the mean ± SD, with three replicates; different lowercase letters indicate significant differences (*p* < 0.05). CK: Control group (distilled water); T0: 150 mM salt treatment; T1: S + 25 μM MT group; T2: S + 50 μM MT group; T3: S + 100 μM MT group; T4: S + 150 μM MT group; T5: S + 200 μM MT group; S: 150 mM salt treatment.

**Table 2:** The effect of exogenous MT (Melatonin) on the growth of cucumber seedlings under salt stress.

Treament	Plant Height/mm	Root Length/mm	Stem Diameter/mm	Leaf Area/mm^2^	Fresh Weight/g	Dry Weight/g
CK	88.67 ± 3.16 ^a^	106.57 ± 4.37 ^b^	3.00 ± 0.26 ^a^	2673.71 ± 393.97 ^b^	3.60 ± 0.19 ^a^	0.98 ± 0.01 ^a^
T0	61.30 ± 2.38 ^c^	94.20 ± 1.57 ^c^	1.70 ± 0.1 ^d^	1410.28 ± 90.17 ^de^	1.56 ± 0.1 ^de^	0.36 ± 0.02 ^ef^
T1	73.30 ± 2 ^b^	105.87 ± 3.91 ^b^	2.43 ± 0.15 ^bc^	1856.65 ± 104.71 ^c^	1.86 ± 0.06 ^cd^	0.52 ± 0.05 ^d^
T2	84.33 ± 3.5 ^a^	120.13 ± 6.64 ^a^	2.80 ± 0.3 ^ab^	3035.82 ± 197.08 ^a^	2.77 ± 0.1 ^b^	0.81 ± 0.06 ^b^
T3	82.27 ± 3.25 ^a^	65.80 ± 1.85 ^d^	2.33 ± 0.15 ^c^	1733.00 ± 55.48 ^cd^	2.12 ± 0.24 ^c^	0.68 ± 0.06 ^c^
T4	72.43 ± 9.36 ^b^	58.57 ± 1.95 ^d^	2.33 ± 0.12 ^c^	1392.82 ± 70.24 ^de^	1.52 ± 0.19 ^e^	0.46 ± 0.1 ^de^
T5	65.53 ± 2.76 ^bc^	47.50 ± 3.36 ^e^	1.83 ± 0.15 ^d^	1183 ± 58.57 ^e^	1.18 ± 0.17 ^f^	0.27 ± 0.03 ^f^

Note: The values represent the mean ± SD, with three replicates; different lowercase letters indicate significant differences (*p* < 0.05). CK: Control group (distilled water); T0: 150 mM salt treatment; T1: S + 25 μM MT group; T2: S + 50 μM MT group; T3: S + 100 μM MT group; T4: S + 150 μM MT group; T5: S + 200 μM MT group; S: 150 mM salt treatment.

**Table 3:** Shows the principal component analysis of all indicators, as well as eigenvalues, contribution rates and cumulative contribution rate.

	Principle Component		
Index	PC1	PC2	PC3
GR	0.29813	−0.2076	0.2715
GP	0.29206	−0.20796	0.27652
GI	0.29428	−0.19856	0.32628
PH	0.3226	0.00539	−0.19108
RL	0.22956	0.15988	−0.58192
LAI	0.23295	0.16228	0.43457
FW	0.31352	−0.12723	−0.21046
DW	0.32954	−0.07845	−0.04268
SP	0.30261	0.16818	−0.07313
SPAD	0.3153	0.05838	−0.17146
MDA	−0.32883	0.05494	0.12315
POD	0.12534	0.49994	0.13346
CAT	0.0674	0.51487	0.25515
SOD	0.0439	0.50023	−0.02094
Eigenvalue	8.66292	3.26506	0.94423
Contribution rate	61.88	23.32	6.74
Cumulative contribution rate	61.88	85.2	91.94

**Table 4:** The comprehensive score of each component through the comprehensive evaluation of the membership function.

Treatment	PC1	PC2	PC3	X_1_(PC1)	X_2_(PC2)	X_2_(PC3)	Dvalue	Comprehensive Sorting	Key Physiological Manifestations
CK	0.336	0.859	1.766	0.580	0.854	1.000	0.650	3	The uncoerced natural state
T0	−1.408	0.388	−1.050	0.000	0.693	0.000	0.076	7	Baseline damage state under salt stress
T1	0.837	−0.437	−0.745	0.747	0.409	0.108	0.666	2	Excellent germination index, moderate injury mitigation
T2	1.596	0.280	−0.764	1.000	0.655	0.102	0.900	1	The biomass is the largest, and the enzyme activity is the highest
T3	−0.290	−1.627	0.798	0.372	0.000	0.656	0.358	5	Abnormal root length inhibition (RLI35%)
T4	−0.533	−0.744	−0.041	0.291	0.304	0.358	0.300	6	The overall effect is the weakest
T5	−0.539	1.282	0.036	0.289	1.000	0.386	0.378	4	Recovery of certain indicators in high-concentration areas

## Data Availability

The original data and experimental protocols can be made public to the scientific community for replication.

## Supplementary Materials

The following supporting information can be downloaded at: https://www.mdpi.com/article/10.3390/life15081294/s1, Table S1: The primers of RT-qRCR used in this study.
