# Supplementary material for: Exogenous Melatonin Induces Salt Stress Tolerance in Cucumber by Promoting Plant Growth and Defense System
Source: Life (Basel). 2025 Aug 14;15(8):1294. doi: 10.3390/life15081294 (PMC12387598; doi:10.3390/life15081294)
Supplement: Supplementary file 1 [file life-15-01294-s001.zip › life-3744481-supplementary.pdf]

Table S1 The primers of RT-qRCR used in this study

**Analysis**	**Primer name**	**Sequence(5′-3′)**	**Tm/℃**	**Amplicon size/bp**
CsSOS for qRT-RCR in transgenic cucumber	CsSOS-F<br>CsSOS-R	TCTGGAGAAGGTTGGCCTGTTAG<br>TTCAAGTCGGTCTGAACAGCATC	58<br>58	72
CsNHX for qRT-RCR in transgenic cucumber	CsNHX-F<br>CsNHX-R	TTGGTGGCGGATCATACTTCGG<br>TGCCGGTACACAATCCAATGAC	60<br>60	145
CsHSF for qRT-RCR in transgenic cucumber	CsHSF-F<br>CsHSF-R	AGGCAGCTCAACACTTATGGATTC<br>AGAAGCCTTCGTTAGCAAATTCCC	58<br>58	70
CsDREB for qRT-RCR in transgenic cucumber	CsDREB-F<br>CsDREB-R	TATTCCCGATCCAGAAGTCATG<br>CAAGCATTGAGAGGTAATCTGC	58<br>58	80
Cucumber *CsActin* gene for and qRT-PCR	Actin-F<br>Actin-R	TCGTGCTGGATTCTGGTG<br>GGCAGTGGTGGTGAACAT	60<br>60	161
